# Homozygous knockout of eEF2K alleviates cognitive deficits in APP/PS1 Alzheimer’s disease model mice independent of brain amyloid β pathology

**DOI:** 10.3389/fnagi.2022.959326

**Published:** 2022-09-09

**Authors:** Nicole P. Kasica, Xueyan Zhou, Hannah M. Jester, Caroline E. Holland, Alexey G. Ryazanov, Tom E. Forshaw, Cristina M. Furdui, Tao Ma

**Affiliations:** ^1^Department of Internal Medicine, Gerontology and Geriatric Medicine, Wake Forest University School of Medicine, Winston-Salem, NC, United States; ^2^Department of Pharmacology, Rutgers Robert Wood Johnson Medical School, Piscataway, NJ, United States; ^3^Section on Molecular Medicine, Department of Internal Medicine, Wake Forest University School of Medicine, Winston-Salem, NC, United States; ^4^Department of Physiology and Pharmacology, Wake Forest University School of Medicine, Winston-Salem, NC, United States; ^5^Department of Neurobiology and Anatomy, Wake Forest University School of Medicine, Winston-Salem, NC, United States

**Keywords:** Alzheimer’s disease, protein synthesis, eEF2K, synapses, memory

## Abstract

Maintenance of memory and synaptic plasticity depends on *de novo* protein synthesis, and accumulating evidence implicates a role of dysregulated mRNA translation in cognitive impairments associated with Alzheimer’s disease (AD). Accumulating evidence demonstrates hyper-phosphorylation of translation factor eukaryotic elongation factor 2 (eEF2) in the hippocampi of human AD patients as well as transgenic AD model mice. Phosphorylation of eEF2 (at the Thr 56 site) by its only known kinase, eEF2K, leads to inhibition of general protein synthesis. A recent study suggests that amyloid β (Aβ)-induced neurotoxicity could be associated with an interaction between eEF2 phosphorylation and the transcription factor nuclear erythroid 2-related factor (NRF2)-mediated antioxidant response. In this brief communication, we report that global homozygous knockout of the eEF2K gene alleviates deficits of long-term recognition and spatial learning in a mouse model of AD (APP/PS1). Moreover, eEF2K knockout does not alter brain Aβ pathology in APP/PS1 mice. The hippocampal NRF2 antioxidant response in the APP/PS1 mice, measured by expression levels of nicotinamide adenine dinucleotide plus hydrogen (NADPH) quinone oxidoreductase 1 (NQO1) and heme oxygenase-1 (HO-1), is ameliorated by suppression of eEF2K signaling. Together, the findings may contribute to our understanding of the molecular mechanisms underlying AD pathogenesis, indicating that suppression of eEF2K activity could be a beneficial therapeutic option for this devastating neurodegenerative disease.

## Introduction

Alzheimer’s disease (AD) is an aging-related neurodegenerative disease characterized by profound cognitive impairments and distinct brain pathology including amyloid β (Aβ) deposition and tau hyper-phosphorylation (Holtzman et al., 2012; Association, 2019; Ma, 2021). Currently there is no cure for AD and related dementias (ADRDs) and completed clinical trials targeting AD-correlated brain pathology met with limited success (Herrup, 2015; Liu and Howard, 2021). It is imperative to develop novel therapeutic strategies based on solid mechanistic studies. A substantial body of literature demonstrates that long-term forms of memory and synaptic plasticity requires *de novo* protein synthesis, i.e., mRNA translation (Costa-Mattioli et al., 2009; Buffington et al., 2014; Remaud et al., 2014). Impaired translational capacity and reduced *de novo* protein synthesis have been demonstrated in the brains of AD patients and multiple rodent models of AD (Ma et al., 2013; Beckelman et al., 2016, 2019; Elder et al., 2021).

Protein synthesis is a fundamental and highly regulated cellular process. There are three phases of protein synthesis including initiation, elongation, and termination. Specific translational protein factors are involved in each phase for regulation of protein synthesis under various conditions (Hershey et al., 2019). Initiation phase is usually considered as the rate-limiting step and critical for translational control. Meanwhile, elongation consumes most (> 95%) of the energy and amino acids used in the mRNA translation process (Browne and Proud, 2002; Kenney et al., 2014). Growing evidence implicates an important role of translation elongation control in synaptic and cognitive functions. One translational factor critically involved in the elongation step is eukaryotic elongation factor 2 (eEF2) (Taha et al., 2013, 2020; Beckelman et al., 2019; Ma, 2021; Yang et al., 2021). eEF2 mediates the translocation step of elongation, catalyzing movement of amino-acyl tRNA from the A-site to the P-site within the ribosome *via* GTP hydrolysis (Browne and Proud, 2002; Proud, 2015). When eEF2 is phosphorylated at its Thr 56 site by its only known kinase, eEF2K, general protein synthesis is repressed (Proud, 2015).

Previous studies from our lab reported hyper-phosphorylation of eEF2 in both *post mortem* hippocampal tissue from AD patients and transgenic mouse models (Ma et al., 2014; Jan et al., 2017; Beckelman et al., 2019). Suppression of eEF2K activity *via* pharmacological inhibitors or siRNA method alleviated amyloid β (Aβ)-induced synaptic failure and neurotoxicity (Ma et al., 2014; Jan et al., 2017; Kasica et al., 2022). The study from Jan et al. (2017) suggested the beneficial effects of eEF2K inhibition on Aβ-induced neurotoxicity were mediated *via* the interactions between eEF2K and the transcription factor nuclear erythroid 2-related factor (NRF2) antioxidant pathway. During AD, prolonged cellular stress leads to an increased production of reactive oxygen species (ROS), prompting the NRF2/antioxidant response element (ARE) system. In response to ROS, NRF2 is translocated to the nucleus, where it promotes the production of neuroprotective proteins, including nicotinamide adenine dinucleotide plus hydrogen NAD(P)H: quinone reductase (NQO1) and heme oxygenase-1 (HO-1) (Saha et al., 2020). Previous literature has reported significantly elevated protein levels of NQO1 and HO-1 in the brains of AD human patients compared to age-matched controls, suggesting a dysregulation of this NRF2-mediated antioxidant response (Schipper et al., 1995; Raina et al., 1999; Wang et al., 2000).

Recently, we reported that heterozygous suppression of eEF2K prevented multiple aspects of AD-associated cognitive and synaptic deficits in AD model mice (Beckelman et al., 2019). Interestingly, there was only a partial rescue of spatial learning and memory [assessed by the hidden-platform Morris water maze (MWM) task] in the AD model mice with heterozygous eEF2K knockdown (Beckelman et al., 2019). Such partial rescuing effects could be attributed to an incomplete suppression of eEF2K activity and eEF2 phosphorylation and raises the question whether complete genetic suppression of the kinase may lead to a full restoration of cognitive performance in AD model mice. To address this question, here we knockout eEF2K completely in the APP/PS1 AD model mice and investigated whether AD-related cognitive impairments would be fully reversed.

## Materials and methods

### Mice

All mice were housed at the Wake Forest School of Medicine barrier facility under the supervision of the Animal Research Program, in compliance with the NIH *Guide for the Care and Use of Laboratory Animals*. Mice adhered to a 12-h light/12-h dark cycle, with regular feeding, cage cleaning, and 24-h food and water access. Both male and female mice were used at the age of 12–16 months for experimentation. A total of 51 mice were used for experiments (WT, *n* = 17; APP, *n* = 9; eEF2K^–/–^, *n* = 16; APP/eEF2K^–/–^, *n* = 9). Breeders of the APP/PS1 mice were purchased from the Jackson Laboratory and expressed human transgenes for APP (KM670/671NL) and presenilin-1 (PSEN1 L166P) (Radde et al., 2006). Homozygous eEF2K^–/–^ mice were a generous gift from Dr. Alexey Ryazanov (Rutgers University) and used to generate double mutant APP/PS1/eEF2K^–/–^ animals. APP/PS1 mice were crossbred with the eEF2K^±^ mice to generate littermate groups: wild type (WT), APP/PS1 (APP), eEF2K^±^, and APP/PS1/eEF2K^–±^ double-mutant mice. Further, eEF2K^±^ mice were crossbred with APP/eEF2K^±^ mice to generate the four experimental genotypes: WT, APP, eEF2K^–/–^, and APP/eEF2K^–/–^ double mutant mice. All genotyping was done by polymerase chain reaction (PCR). All protocols involving animals were approved by the Institutional Animal Care and Use Committee of Wake Forest University School of Medicine.

### Western blot

Mouse hippocampal tissue was flash-frozen on dry ice and sonicated as previously described in lysis buffer with protease and phosphatase inhibitors (Ma et al., 2014). Samples containing equal amounts of protein lysate were loaded on 4–12% Tris-glycine SDS-PAGE (Bio-Rad) gels for standard gel electrophoresis. Following the transfer, nitrocellulose membranes were blocked for 10 min in SuperBlock TBS Blocking Buffer (Thermo Fisher Scientific). All primary and secondary antibodies were diluted in 5% milk/TBST or 5% BSA/TBST. Blots were probed with primary antibodies for: eEF2K (1:500, Cell Signaling Technology, catalog 3692), phospho-eEF2 (Thr56) (1:1,000, Cell Signaling Technology, catalog 2331); eEF2 (1:1,000, Cell Signaling Technology, catalog 2332); β-Actin (1:5,000, Sigma Aldrich, catalog A2228); NQO1 (1:1,000, Cell Signaling Technology, catalog 62262); HO-1 (1:1,000, Cell Signaling Technology, catalog 43966); 4HNE (1:500, R&D Systems, Catalog MAB3249); phospho-tau (Ser396) (1:1,000, Thermo Fischer, catalog 44-752G), tau (1:1,000, Sigma-Aldrich, catalog T5530), GAPDH (1:10,000, Cell Signal, catalog 2118). Following primary antibody incubation, secondary antibodies were applied, either goat anti-rabbit IgG (H + L)-HRP conjugate (1:5,000, Bio-Rad, catalog 170-6515) or goat anti-mouse IgG (H + L)-HRP conjugate (1:5,000, Bio-Rad, catalog 170-6516). Proteins were visualized using the ChemiDoc Imaging System (Bio-Rad). Densitometric analysis was performed using ImageJ software (NIH). Phospho-proteins were normalized to levels of total protein, and total proteins were normalized using housekeeping protein GAPDH.

### Mouse tissue immunohistochemistry

Following euthanasia *via* cervical dislocation, mouse brains were hemisected and fixed overnight in ice-cold PFA and transferred to 70% EtOH. Paraffin-embedding was performed by Wake Forest Pathology core service. Paraffin-embedded sections (5 μm) mounted on charged slides were cleared in xylene and rehydrated through a graded ethanol series. Sections were pre-treated in boiling citrate buffer for 10 min, and were blocked for 2 h with 10% NGS in 1% BSA/TBS. Slides were incubated in primary antibody for amyloid-β (6E10) (mouse monoclonal; 1:200; BioLegend, catalog 803001) or 4HNE (mouse monoclonal, 1:250, R&D Systems, catalog MAB3249) in a humidified chamber overnight at 4°C. Following a 15-min block in 3% hydrogen peroxide, sections were incubated in biotinylated anti-mouse secondary antibody (1:200, Vector Labs, catalog BA-2000) for 1 h at room temperature, followed by Vectastain Elite ABC Reagent (Vector Labs, catalog PK-6100) for 30 min. Primary and secondary antibodies as well as ABC reagent were diluted in 1% BSA/TBS. Sections were developed in ImmPACT DAB Substrate Kit, Peroxidase (Vector Labs, catalog SK-4105) for 30 s to 3 min with monitoring. Slides were counterstained using Mayer’s hematoxylin for 60 s and stained blue with 0.2% lithium carbonate for 20 s. In between each step, sections were rinsed using distilled water or TBSTX (pH 7.4). Negative controls were incubated in 1% BSA with no primary antibody. Sections were dehydrated in an alcohol series and cleared with xylene, coverslipped, and dried overnight. Slides were imaged at 2X, 20X, and 60X on a Keyence BZ-X710 All-in-One Fluorescent Microscope (Keyence). Densitometric analysis was performed using 2X and 20x images and ImageJ software. For 4HNE, the number of positive cells was compared to total number of nuclei present within a given region of interest (ROI) to get percentage of positive cells per ROI.

### Amyloid β ELISA

Frozen mouse forebrain samples were sonicated as previously described (Ma et al., 2014). Samples were centrifuged at 16,000 × g for 20 min at 4°C, and the supernatant was collected for ELISA. Aβ 1-42 (Thermo Fisher Scientific, catalog KMB3441) and Aβ 1–40 (Thermo Fisher Scientific, catalog KMB3481) ELISAs were performed according to the manufacturer’s instructions. 96-well plates were read at 450 nm using an iMark™ microplate reader (Bio-Rad).

## Mouse behavioral assays

### Open field assay

Mice were handled for at least 5 days prior to behavioral testing and habituated to the testing facility for an hour prior to experimentation. Animals were placed in an opaque plastic open field (OF) chamber (40 cm × 40 cm × 40 cm) and allowed to explore for 15 min. Time spent in the center and periphery of the chamber was measured and calculated as a percent of total time. Distance moved and velocity was measured using Ethovision XT tracking software (Noldus Information Technology, Leesburg, VA). Data collection and analysis were performed blinded.

### Novel object recognition

Mice underwent a 2-day familiarization protocol in which they were placed in an opaque, plastic arena (40 cm × 40 cm × 40 cm) with 2 identical objects and allowed to explore for 5 min. Twenty-four hours after familiarization, animals were tested in the arena for 5 min with one object replaced with a novel object. All objects were randomly assigned to mice, and the placement of novel objects was counterbalanced. Time spent with each object was measured and calculated as a ratio of the total interaction time. A novel object preference ratio of less than 0.5 indicates less than 50% of total time was spent with the novel object and shows memory impairment. Time with objects was measured both manually and using EthoVision XT tracking software. Mice with a total interaction time of fewer than 10 s were excluded from the analysis. Data collection and analysis were performed blinded.

### Morris water maze

Morris Water Maze was performed as previously described (Ma et al., 2013). Briefly, animals were trained to find an escape platform (10 cm diameter) submerged in an opaque pool of water (135 cm diameter) surrounded by visuospatial cues. The escape platform was covered by 2 cm of water containing non-toxic white paint. Training consisted of 4 trials (60 s maximum, 15 min intertrial interval) per day for 5 consecutive days. Escape latency was measured each training day. A probe trial was performed 2 h following training on the fifth day. The visible maze platform task consisted of 4 trials per day for 2 consecutive days with the escape platform marked by a visible cue and moved randomly between four locations. Trajectories, time spent in each maze quadrant, velocity, and distance moved were recorded using Ethovision XT software. Data collection and analysis were performed blinded.

### Data analysis

Data are presented as mean + SEM. Summary data are presented as group means with SE bars. For comparisons between two groups, a two-tailed independent Student’s *t*-test was performed using Prism 6 statistics software (GraphPad Software, San Diego, CA). For comparisons between more than two groups, one-way ANOVA was used with Tukey’s *post-hoc* tests for multiple comparisons. Error probabilities of *p* < 0.05 were considered statistically significant.

## Results

### Knockout of eEF2K ameliorates Alzheimer’s disease-associated eukaryotic elongation factor 2 hyper-phosphorylation without affecting amyloid pathology

**FIGURE 1:**
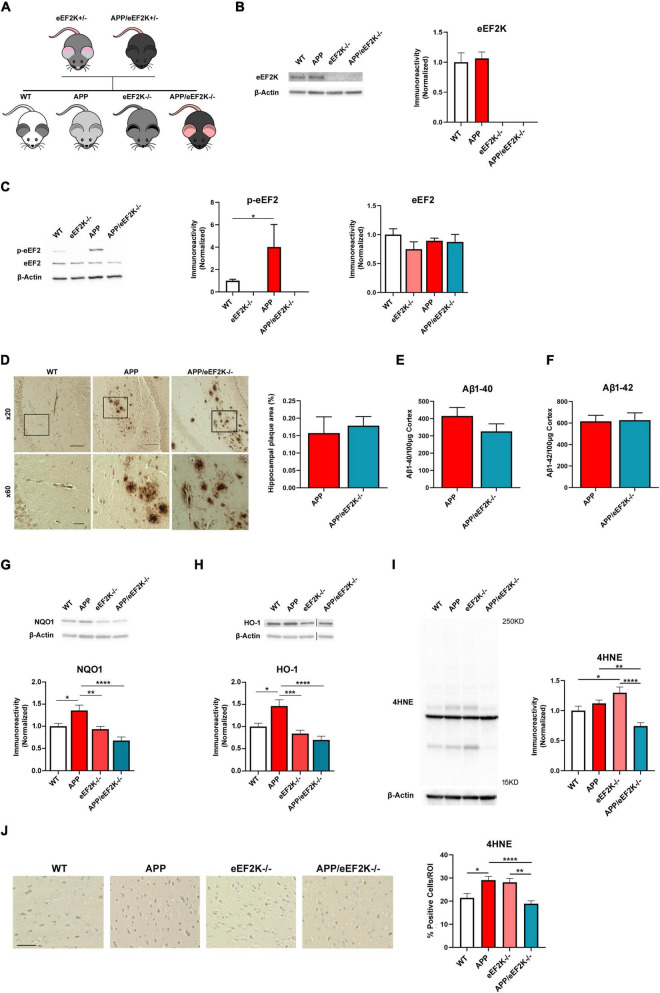
Homozygous eEF2K knockout alleviates AD-associated eEF2 hyper-phosphorylation and dysregulated NRF2-mediated antioxidant response without affecting amyloid β (Aβ) pathology in APP/PS1 mice. **(A)** Breeding schematic showing generation of APP/eEF2K^–/–^ double mutant mice. **(B)** Western blot showing eEF2K levels in hippocampal lysates. eEF2K levels depleted in eEF2K^–/–^ and APP/eEF2K^–/–^ mice (WT, *n* = 4; APP, *n* = 4; eEF2K^–/–^, *n* = 3; APP/eEF2K^–/–^, *n* = 4) **(C)** Western blot showing p-eEF2 and eEF2 levels in hippocampal lysates. Levels of phosphorylated eEF2 are significantly lower in eEF2K^–/–^ mice than WT mice and are significantly lower in APP/eEF2K^–/–^ mice than APP (WT, *n* = 6; APP, *n* = 5; eEF2K^–/–^, *n* = 4; APP/eEF2K^–/–^, *n* = 6). WT vs. eEF2K^–/–^, *P < 0.0001; APP vs. APP/eEF2K^–/–^, *p = 0.03. No differences in levels of total eEF2 were detected. **(D)** Representative images and quantification of hippocampal plaque deposition in WT, APP, and APP/eEF2K^–/–^ mice. Insets are shown at 60X magnification. Scale bars: 100 μm (x20); 20 μm (x60). No differences in percentage of hippocampal area covered in amyloid plaques in APP (*n* = 10 sections), and APP/eEF2K^–/–^ (*n* = 8) were detected. **(E)** ELISA showed no differences in levels of Aβ 1–40 or **(F)** Aβ 1–42 in APP and APP/eEF2K^–/–^ forebrain tissue (APP, *n* = 6; APP/eEF2K^–/–^, *n* = 6). **(G)** Western blot showing NQO1 levels in hippocampal lysates. Levels of NQO1 significantly increased in APP mice compared to WT, eEF2K^–/–^, and APP/eEF2K^–/–^ mice (WT, *n* = 6; APP, *n* = 5; eEF2K^–/–^, *n* = 6; APP/eEF2K^–/–^, *n* = 6). WT vs. APP, *p = 0.0268; APP vs. eEF2K^–/–^, *p = 0.0065; APP vs. APP/eEF2K^–/–^, *p < 0.0001, 1-way ANOVA with Tukey’s *post-hoc* test, *F* = 10.63. **(H)** Western blot showing HO-1 levels in hippocampal lysates. Levels of HO-1 significantly increased in APP mice compared to WT, eEF2K^–/–^, and APP/eEF2K^–/–^ mice (WT, *n* = 5; APP, *n* = 5; eEF2K^–/–^, *n* = 6; APP/eEF2K^–/–^, *n* = 6). WT vs. APP, *p = 0.0103; APP vs. eEF2K^–/–^, *p = 0.0002; APP vs. APP/eEF2K^–/–^, *p < 0.0001, 1-way ANOVA with Tukey’s *post-hoc* test, *F* = 11.47. **(I)** Representative blot and quantification graph showing 4HNE levels in hippocampal lysates. Levels of 4HNE significantly decreased in APP/eEF2K-/- mice compared to APP and eEF2K^–/–^ mice (WT, *n* = 7; APP, *n* = 8; eEF2K^–/–^, *n* = 8; APP/eEF2K^–/–^, *n* = 8). WT vs. eEF2K^–/–^, *p = 0.0430; APP vs. APP/eEF2K^–/–^, **p = 0.0051; eEF2K^–/–^ vs. APP/eEF2K^–/–^, ****p < 0.0001, 1-way ANOVA with Tukey’s *post-hoc* test, *F* = 11.28. **(J)** Representative images and graph showing percentage of 4HNE positive cells. Images are 20x magnification. Scale bar = 50μm. Percentage of 4HNE positive cells significantly decreased in APP/eEF2K^–/–^ mice compared to APP and eEF2K^–/–^ mice (WT, *n* = 3; APP, *n* = 3; eEF2K^–/–^, *n* = 3; APP/eEF2K^–/–^, *n* = 3) 3 sections per sample and 10–15 images per section. WT vs. APP, *p = 0.0228; APP vs. APP/eEF2K^–/–^, ****p < 0.0001; eEF2K^–/–^ vs. APP/eEF2K^–/–^, **p < 0.0015, 1-way ANOVA with Tukey’s *post-hoc* test, *F* = 8.689.

To investigate whether a complete suppression of eEF2 phosphorylation could alleviate AD pathophysiology, a genetic approach was used to knockout eEF2K completely in AD by crossing the eEF2K ± mice with APP/eEF2K ± mice as described in the method section. Briefly, four experimental groups were generated including: wild type (WT), APP/PS1 (APP), eEF2K^–/–^, and APP/PS1/eEF2K^–/–^ double mutant (APP/eEF2K^–/–^) (Figure 1A). Levels of eEF2K were depleted in the eEF2K-/- and APP/eEF2K-/- mice (Figure 1B). Consistent with previous studies (Beckelman et al., 2019), levels of eEF2 phosphorylation in hippocampal lysates were increased in APP mice compared to WT littermates (Figure 1C). Knockout of eEF2K drastically reduced eEF2 phosphorylation in APP mice (Figure 1C) without affecting protein levels of total eEF2 (Figures 1B,D).

To determine the effects of eEF2K knockout on AD-associated brain pathology, we first examined amyloid beta (Aβ) pathology with an immunohistochemical approach and found similar levels of Aβ plaque deposition in the hippocampi of APP and APP/eEF2K^–/–^ mice (Figure 1D). We also utilized ELISA to measure levels of brain Aβ 1-40 and Aβ 1-42 in the cortex and found no differences between APP and APP/eEF2K^–/–^ mice (Figures 1E,F). Furthermore, genetic knockout of eEF2K did not affect levels of phosphorylated tau in APP and APP/eEF2K^–/–^ mice (Supplementary Figure 1). Together, these findings reveal that genetic knockout of eEF2K abolishes eEF2 phosphorylation but does not affect Aβ or tau pathology in the APP/PS1 AD model mice.

We next investigated the effect of eEF2K knockout on the NRF2-mediated antioxidant response. Consistent with previous studies in *post mortem* human AD brain samples (Schipper et al., 1995; Raina et al., 1999; Wang et al., 2000), we found that protein levels of NQO1 and HO-1 were significantly increased in the hippocampi of the APP mice compared to the WT group (Figures 1G,H). Notably, levels of NQO1 and HO-1 expression in hippocampi of the APP/eEF2K-/- mice were similar to those in the WT mice (Figures 1G,H), suggesting the genetic suppression of eEF2 phosphorylation ameliorated AD-related dysregulation of the NRF2-mediated antioxidant response. In addition, ROS levels were measured using 4-hydroxynonenal (4HNE). 4HNE is a byproduct of lipid peroxidation and is a valid marker of ROS levels (Jaganjac et al., 2020). In agreement with NRF2 signaling, 4HNE levels were significantly decreased in the APP/eEF2K-/- mice compared to both APP and eEF2K-/- mice using both Western blot and immunohistochemistry approaches (Figures 1I,J).

### Knockout of eEF2K alleviates long-term recognition memory deficits in the APP/PS1 mice

**FIGURE 2:**
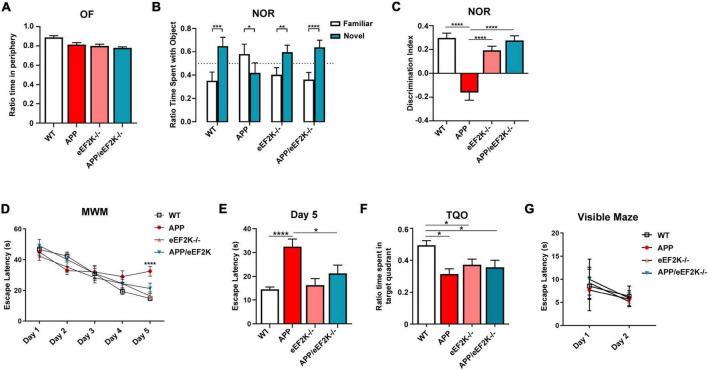
Genetic suppression of eEF2K prevents AD-associated cognitive deficits in APP mice. **(A)** Ratio time spent in periphery in the OF task (WT, *n* = 17; APP, *n* = 9; eEF2K^–/–^, *n* = 10; APP/eEF2K^–/–^, *n* = 8). **(B)** Ratio time spent with familiar (white) and novel (blue) objects in the NOR task during testing phase (WT, *n* = 17; APP, *n* = 9; eEF2K^–/–^, *n* = 10; APP/eEF2K^–/–^, *n* = 8). Statistical preference for novel object: WT, ***p = 0.0002; APP, *p* = 0.822; eEF2K^–/–^, **p = 0.0014; APP/eEF2K^–/–^, ****p < 0.0001, unpaired *t*-tests. **(C)** Discrimination index [(time spent exploring novel object – time spent exploring familiar object)/total exploration time] (WT, *n* = 17; APP, *n* = 9; eEF2K^–/–^, *n* = 10; APP/eEF2K^–/–^, *n* = 8). WT vs. APP, ****p < 0.0001; APP vs. eEF2K^–/–^, ****p < 0.0001; APP vs. APP/eEF2K^–/–^, ****p < 0.0001, 1-way ANOVA with Tukey’s *post hoc*-test, *F* = 23.99. **(D)** Escape latency (s) over 5 days of training in the hidden platform MWM. Four trials/day (WT, *n* = 16; APP, *n* = 9; eEF2K^–/–^, *n* = 16; APP/eEF2K^–/–^, *n* = 9). WT vs. APP, ****p < 0.0001; APP vs. APP/eEF2K^–/–^, *p = 0.04, 1-way ANOVA with Tukey’s *post-hoc* test, *F* = 8.888. **(E)** Escape latency (s) on day 5 of MWM training (WT, *n* = 16; APP, *n* = 9; eEF2K^–/–^, *n* = 16; APP/eEF2K^–/–^, *n* = 9). WT vs. APP, ****p < 0.0001; APP vs. APP/eEF2K^–/–^, *p = 0.04, 1-way ANOVA with Tukey’s *post-hoc* test, *F* = 8.888. **(F)** Ratio time spent in target quadrant during probe trial phase of MWM (WT, *n* = 16; APP, *n* = 9; eEF2K^–/–^, *n* = 16; APP/eEF2K^–/–^, *n* = 9). **(G)** Escape latency (s) over 2 days in the visible maze task (WT, *n* = 16; APP, *n* = 9; eEF2K^–/–^, *n* = 16; APP/eEF2K^–/–^, *n* = 9).

Next, we subjected the mice to a series of behavioral tasks to assess their cognitive performances. We first performed the OF task to assess baseline anxiety-like behavior and locomotor activity. We observed no significant differences among the four genotypes in OF task, including time spent in the peripheral area, travel distance, and velocity (Figure 2A and Supplementary Figure 2A). Furthermore, we performed the novel object recognition (NOR) task to evaluate long-term recognition memory (Zimmermann et al., 2020). WT mice exhibited a preference for the novel over the familiar object as assessed by the ratio time spent with the objects and the discrimination index (Figures 2B,C). In contrast, APP mice did not show a preference for the novel object, indicating a cognitive deficit (Figures 2B,C). Interestingly, APP mice spent more time interacting with the familiar object (Figures 2B,C). Importantly, APP/eEF2K^–/–^ mice performed similarly to WT mice, spending more time with the novel than the familiar objects (Figures 2B,C). Additionally, eEF2K-/- mice demonstrated normal recognition memory, interacting more with the novel object (Figures 2B,C). Thus, eEF2K knockout prevented impairment of long-term recognition memory in the APP/PS1 AD model mice.

Moreover, we used the hidden-platform MWM task to assess spatial learning and memory (Zimmermann et al., 2020; Kasica et al., 2022). Both WT and eEF2K^–/–^ mice exhibited normal learning during the training, indicated by marked day-to-day decreases in escape latency during the acquisition phase (Figures 2D,E). Consistent with previous studies (Zimmermann et al., 2020; Kasica et al., 2022), APP mice demonstrated longer escape latency, indicating a cognitive impairment (Figures 2D,E). Importantly, impaired learning observed in the APP mice was alleviated in the APP/eEF2K^–/–^ mice, as demonstrated by improved day-to-day escape latency (Figures 2D,E). In agreement with previous studies, APP mice showed impaired spatial memory assessed by the target quadrant occupancy (TQO) during the probe trial (Figure 2F). To our surprise, the TQO of the APP/eEF2K^–/–^ mice is indistinguishable from that of the APP mice, and significantly lower than the TQO of the WT mice (Figure 2F). Such findings indicate that eEF2K knockout failed to improve AD-associated spatial memory impairment. Interestingly, eEF2K^–/–^ mice showed deficits in spatial memory, as indicated by lower TQO compared to WT mice (Figure 2F). Distance traveled and velocity of movement during the probe trial were unaltered across the four genotypes (Supplementary Figure 2B). We also examined potential learning and memory-independent effects of eEF2K knockout (e.g., swimming ability, vision, and motivation) through the visible maze task (Zimmermann et al., 2020; Kasica et al., 2022). No differences in latency to locate the visible platform were observed across all the genotypes (Figure 2G). Taken together, genetic eEF2K knockout improved spatial learning but not spatial memory deficits in the APP/PS1 AD model mice.

## Discussion

Concurrent with aging and without effective treatments available, AD has become a global threat to public health. It is urgent to identify novel therapeutic targets based on solid mechanistic studies. Here, we show that homozygous genetic knockout of the eEF2K gene led to a restoration of long-term recognition memory in the aged APP/PS1 AD model mice (Figures 2B,C). We previously showed that heterozygous reduction of eEF2K partially improved spatial learning and memory deficits in an AD mouse model (Beckelman et al., 2019), leading to the hypothesis that further suppression (e.g., homozygous knockout) of eEF2K and eEF2 phosphorylation would fully restore the impairments of spatial learning and memory. However, results from the MWM experiments demonstrated that while eEF2K knockout can improve spatial learning, it does not alleviate spatial memory deficits in the AD mice (Figures 2D–F). Previous studies with young (<2 months) eEF2K^–/–^ mice reported normal spatial learning and memory during the MWM test (Heise et al., 2017). In contrast, older eEF2K-/- mice in the current study (12–16 months old) displayed normal recognition memory (Figures 2B,C) but impaired spatial memory (Figure 2F). We recently showed impairments of recognition memory in aged mice (>20 months) were prevented by eEF2K knockout. Interestingly, suppression of eEF2K failed to improve aging-related spatial learning and memory (Gosrani et al., 2020). The integral hippocampus is vital for rodents to perform both behavioral tasks (i.e., NOR and MWM). Meanwhile, other brain regions such as the prefrontal cortex (PFC) are likely involved in these cognitive functions and may play distinct roles (Akirav and Maroun, 2006; Buzsáki and Moser, 2013). It would be intriguing in future studies to elucidate the roles of eEF2K/eEF2 signaling in memory consolidation process under different paradigms, with the aging factor taken into consideration. While this study utilized both male and female mice it is unclear if sex may be an underlying factor in our results and this could be an avenue for future studies.

Previous studies indicate that upregulation of translation initiation through manipulations of translation initiation factors (e.g., eIF2) could improve multiple aspects of AD pathophysiology including cognitive and synaptic deficits (Ma et al., 2013; Oliveira et al., 2021). These findings support the concept that a boost of general protein synthesis, through regulation of either initiation and/or elongation process, could be a potential therapeutic strategy for AD and related dementias. In comparison to the “partial” alleviation of AD-related cognitive deficits in the current study, the aforementioned studies targeting the translation initiation mechanisms *via* genetic or pharmacological approaches showed a complete restoration of the spatial memory function in the aged APP/PS1 AD model mice (Ma et al., 2013; Oliveira et al., 2021). Future studies to elucidate the detailed molecular mechanisms underlying such differences are warranted.

Suppression of eEF2 phosphorylation by targeting eEF2K could be an attractive therapeutic strategy for AD from several perspectives. First, eEF2K belongs to the “alpha-kinases” (α-kinases) family, which includes only 6 members in the human genome (Drennan and Ryazanov, 2004). Importantly, the catalytic domains of the α-kinases are distinct from those in the conventional protein kinases (CPKs), which consist of the vast majority of the eukaryotic protein kinases possessing homologous catalytic domains (e.g., serine/threonine kinases and tyrosine kinases). Therefore, small molecule antagonists of eEF2K (if designed appropriately to target the catalytic domains unique to the α-kinases) are unlikely to interfere with the activities of CPKs, which are critically involved in a broad spectrum of biological processes (Drennan and Ryazanov, 2004; Liu and Proud, 2016; Ma, 2021). Moreover, substantial evidence indicates that eEF2K activity is not required for development or cell survival under physiological conditions. Transgenic mice with global knockout of eEF2K or wild-type mice treated with the chronic treatment of eEF2K inhibitors appear normal in numerous measurements of basic biological functions during development (Heise et al., 2017; Ma, 2021; Kasica et al., 2022). Thus, eEF2K could be a “safe” target, which is important for AD patients who usually need to take medicine continuously over a long period. Finally, eEF2K is the only known upstream kinase for eEF2 (Thr56 phosphorylation) (Proud, 2015). Until recently, eEF2 was the only known downstream target of eEF2K. However, the use of new mass spectrometry techniques has determined novel substrates of eEF2K, namely the alpha4 subunit of protein phosphatase 2A (PP2A) and the alpha subunit of AMP-activated protein kinase (AMPK) (Lazarus et al., 2017). Interestingly, both of these novel targets have also been implicated in AD. Alpha4 is responsible for regulating PP2A activity by preventing its degradation (Kong et al., 2009; LeNoue-Newton et al., 2011). Tau hyperphosphorylation and aggregation is a classical hallmark of AD pathology. Notably, PP2A regulates tau phosphorylation by dephosphorylating tau as well as multiple tau kinases (Martin et al., 2013). PP2A activity and expression has been shown to be decreased in AD brains (Sontag et al., 2004; Liu et al., 2005). The second novel substrate, AMPK has also been implicated in AD and extensive work has shown that AMPK alpha isoforms are dysregulated in AD and have distinct roles in its pathogenesis (Wang et al., 2019, 2021; Zimmermann et al., 2020). The potential regulation of PP2A and AMPK by eEF2K increases its validity as a therapeutic target in AD.

Recent literature has shown that eEF2K suppression could be an effective strategy to rescue AD-associated neuronal dysfunction. Upon exposure to cellular stressors including H_2_O_2_ and Aβ42, neuronal cells with eEF2K siRNA knockdown (eEF2K kd) displayed higher cellular viability (Jan et al., 2017). The increased survival can be attributed to eEF2K’s interactions with the NRF2/ARE mechanism that promotes the transcription of neuroprotective proteins in response to ROS. Under normal physiological conditions, ROS prompt the translocation of NRF2 to the nucleus, where it preferentially increases the expression of major ROS detoxifying mediators including NQO1 and HO-1 (Harder et al., 2015). Interestingly, in AD nuclear NRF2 levels are decreased compared to age-matched controls (Ramsey et al., 2007), but NQO1 and HO-1 are significantly overexpressed in the hippocampi of *post mortem* AD brains (Schipper et al., 1995; Raina et al., 1999; Wang et al., 2000). Transient increase of NQO1 and HO-1 can have beneficial effects under acute ROS exposure, acting as antioxidants and promoting cellular survival (Si et al., 2020; Ross and Siegel, 2021). However, chronic cellular stress, as is observed in AD, leads to long-lasting overexpression of NQO1 and HO-1 in the hippocampus, resulting in potential detrimental effects including neuroinflammation, cognitive deficits, impaired synaptic plasticity, and decreased expression of memory-related proteins (Gupta et al., 2014; Li et al., 2015; Wang et al., 2015; Ross and Siegel, 2021). In the current study, we show that eEF2K knockout alleviates the AD-associated overexpression of NQO1 and HO-1 in AD model mice (Figures 1G,H), suggesting the beneficial effects observed in the double mutant mice may be attributed to restored homeostasis of the NRF2-mediated antioxidant response.

In summary, this study expands upon previous work from our lab exploring the roles of the eEF2K/eEF2 signaling in AD. Taken together, these studies suggest that eEF2K suppression confers protective effects against cognitive deficits displayed in aged and AD model mice (Beckelman et al., 2019; Gosrani et al., 2020). Thus, the eEF2/eEF2K pathway may be an alluring target for novel pharmacological interventions for AD, but the level to which eEF2K is inhibited to produce a therapeutic effect will need to be further explored.

## Data availability statement

The raw data supporting the conclusions of this article will be made available by the authors, without undue reservation.

## Ethics statement

This animal study was reviewed and approved by the Institutional Animal Care and Use Committee of Wake Forest University School of Medicine.

## Author contributions

NK conceptualized experiments, collected and analyzed the data, and wrote the manuscript. XZ, HJ, CH, TF, and CF collected the data and analyzed the data. AR provided the eEF2K^–/–^ mice. TM conceptualized experiments and wrote the manuscript. All authors contributed to the article and approved the submitted version.

## Supplementary material

Supplementary Figure 1 Levels of tau phosphorylation are not affected by genetic eEF2K knockout. Representative Western blots and quantification graphs showing levels of phosphorylated tau (Ser396) and total tau in hippocampal lysates from the 4 genotypes. No significant differences in levels of phosphorylated tau (Ser396) were detected across the 4 genotypes No significant differences in levels of total tau were detected (WT, *n* = 8; APP, *n* = 8; eEF2K^–/–^, *n* = 6; APP/eEF2K^–/–^, *n* = 5).

Supplementary Figure 2 Control behavioral data for APP/eEF2K^–/–^ cohorts. **(A)** Left: Distance traveled in cm in the OF assay. Right: Velocity of movement in cm/s in the OF assay (WT, *n* = 17; APP, *n* = 9; eEF2K^–/–^, *n* = 10; APP/eEF2K^–/–^, *n* = 8). **(B)** Left: Distance traveled in cm in the MWM probe trial (WT, *n* = 16; APP, *n* = 9; eEF2K^–/–^, *n* = 16; APP/eEF2K^–/–^, *n* = 9). Right: Velocity of movement in cm/s in the MWM probe trial.
