# Supplementary figures and images for: Homozygous knockout of eEF2K alleviates cognitive deficits in APP/PS1 Alzheimer’s disease model mice independent of brain amyloid β pathology
Source: Front Aging Neurosci. 2022 Sep 9;14:959326. doi: 10.3389/fnagi.2022.959326 (PMC9500344; doi:10.3389/fnagi.2022.959326)

Figure S1

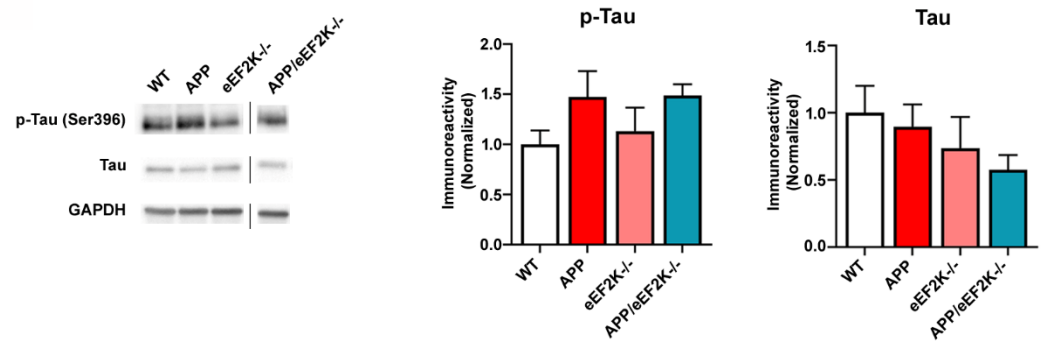

Figure S2

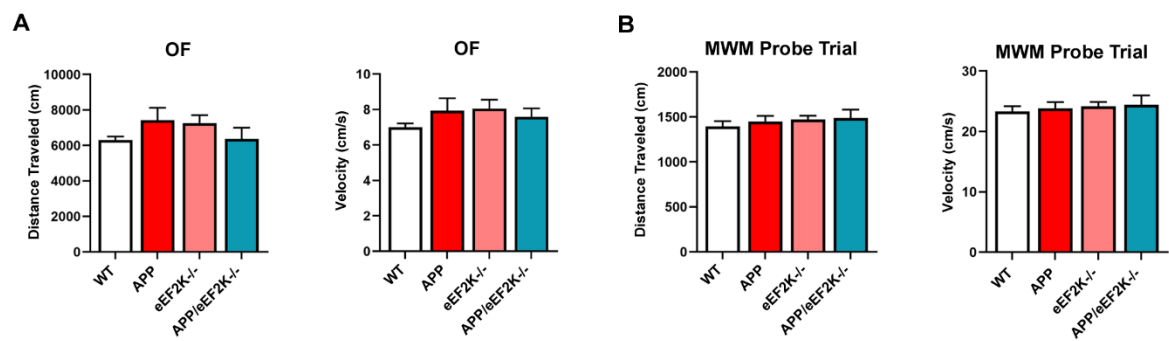

Supplement: Supplementary Figure 1 — Levels of tau phosphorylation are not affected by genetic eEF2K knockout. Representative Western blots and quantification graphs showing levels of phosphorylated tau (Ser396) and total tau in hippocampal lysates from the 4 genotypes. No significant differences in levels of phosphorylated tau (Ser396) were detected across the 4 genotypes No significant differences in levels of total tau were detected (WT, n = 8; APP, n = 8; eEF2K–/–, n = 6; APP/eEF2K–/–, n = 5). [file Image_1.pdf]
